# Phase transitions and asymmetry between signal comprehension and production in biological communication

**DOI:** 10.1038/s41598-019-40141-4

**Published:** 2019-03-05

**Authors:** Mohammad Salahshour, Shahin Rouhani, Yasser Roudi

**Affiliations:** 1 Department of Physics, Sharif University of Technology, P.O. Box 11165-9161 Tehran, Iran; 2 Kavli Institute for Systems Neuroscience and Centre for Neural Computation, Norwegian University of Science and Technology (NTNU), Olav Kyrres gate 9, 7030 Trondheim, Norway

## Abstract

We introduce a model for collective information acquisition from the environment, in a biological population. In this model, individuals can make noisy observations of the environment, and communicate their observation by production and comprehension of signals. As the communication noise decreases, the model shows an order-disorder transition from a disordered phase in which no consensus about the environmental state exists to an ordered phase where the population forms a consensus about the environmental state. The ordered phase itself is composed of an informed consensus, in which the correct belief about the environment prevails, and an uninformed consensus phase, in which consensus on a random belief about the environmental state is formed. The probability of reaching informed consensus increases with increasing the observation probability. This phenomenology implies that a maximum noise level, and a minimum observation probability are necessary for informed consensus in a communicating population. Furthermore, we show that the fraction of observant individuals needed for the group to reach informed consensus decreases with increasing population size. This results from a shift in the uninformed-informed transition to smaller observation probabilities by increasing population size. Importantly, we also find that an amount of noise in signal production deteriorates the information flow and the inference capability, more than the same amount of noise in comprehension. This finding implies that there is higher selection pressure to reduce noise in production of signals compared to comprehension. Regarding this asymmetry, we propose an experimental design to separately measure comprehension and production noise in a given population and test the predicted asymmetry.

## Introduction

Information acquisition about environmental conditions is vital for populations living in an uncertain environment^1–6^. One of the most astonishing ways to accomplish this task is collective information acquisition by communication between individuals in a population, by exchanging signals^6–8^. Examples of such populations can be found allover the biological world, from microbial organisms to large animals. Bacterial populations exchange information about environment in what is known as quorum sensing^9,10^. Communication between cells in multicellular organisms, is vital for the functioning of the cell and the organism as whole^11^. Social and eusocial insects use different sorts of signals, from visual, acoustic, to chemical and tactile signals, to communicate over diverse issues vital for survival of the group^12,13^. In many large group living animals^14^, individuals use signals to exchange information. Finally, human language can be thought of as a highly evolved version of such signaling communication systems^15–17^. As many other biological systems, communication systems generally operate under noisy conditions and are prone to errors in signal production and comprehension. Eliminating noise is typically far from feasible, and lowering noise is subject to costs and constraints^18^. An important challenge for biological populations is therefore; how to achieve highest information acquisition capabilities, given the constraints on lowering noise in signal production and comprehension?

To shed light on this question, and on the mechanism by which a population collectively extracts information from the environment, we introduce a model in which individuals use three communication channels for observation of the environment, and sharing their information by production and comprehension of signals. The model shows an order-disorder transition from a consensus phase at low communication noise to a disordered phase in which no consensus is formed, at high communication noise. The ordered phase itself is composed of two phases: The informed consensus phase, in which the population collectively forms the correct belief about the environmental state, and an uninformed consensus phase, in which consensus is formed on a random belief about the environmental state. Transition from uninformed consensus to informed consensus is discontinuous and happens when the flow of information to the population through direct observations by individuals increases. This shows that for communication to increase the information acquisition capability of a population, a high enough information flow to the population through direct observations, and a low enough communication noise is necessary. Furthermore, we show that, the fraction of observant individuals needed for the population to correctly infer the environmental state, decreases with the population size. This results from a shift in the uninformed-informed transition line to smaller observation probabilities by increasing the population size, due to finite size effects. Finally, we discover a fundamental asymmetry between signal production and comprehension. We show that noise in signal production is more detrimental than noise in signal comprehension, because it disrupts the flow of information between individuals more than the later, and decreases the amount of information that the population can collectively reach from environment. This finding, predicts that signal production channels of communication systems, from bacteria to human language, should have been under higher selection pressure for noise reduction, and we should observe higher levels of regulation on signal production faculties compared to signal comprehension. We propose an experimental set-up to separately measure noise in comprehension and in production of signals in a population and test the asymmetry predicted here.

## The model

We consider a population of *N* communicating individuals living in an environment which can take one out of *n* possible states. Without loss of generality, we assume the population resides on a communication network, such that each individual when intending to signal others, transmits its signals to its neighbors on the network.

Our model of collective information acquisition has three ingredients. First, we model individuals’ abilities to a) observe the environment, b) to produce signals, and c) to comprehend signals, by introducing three communication channels. Second, individuals make inferences based on the information they have received. For this purpose they need a decision making mechanism. And third, the model introduces a dynamics for communication between individuals. Below, we introduce each part of the model separately.

### Collective sensing system_

Individuals have access to *n* representations, each corresponding to one of the environmental states, and can communicate their representation, by using *n* signals, each corresponding to one of the representations. Generally, observation of the environment, signal production, and comprehension are subject to errors and are done probabilistically. We implement this fact by introducing three conditional probabilities, or communication channels that individuals have for observation of the environment, production of signals, and comprehension of signals: the representation matrix $R(r|\varepsilon )$, gives the conditional probability that as a result of observing environmental state $\varepsilon$, representation *r* is produced. In the same way, the production matrix $G(\sigma |r)$, is used for signal production and gives the conditional probability that signal *σ* is produced for representation *r*, and the comprehension matrix $C(r|\sigma )$, is used for comprehension of signals, and gives the conditional probability that representation *r* is comprehended, when receiving signal *σ*. As mentioned earlier, in biological settings, all these activities are subject to noise. We incorporate noise into the model by parameterizing these matrices with a noise parameter ${\eta }_{x}$, where *x* can be *R*, *G* and *C* referring to the different channels of communication we just defined. We take the diagonal elements to be $1\,-\,{\eta }_{x}$, and off-diagonal elements to be $\frac{{\eta }_{x}}{n\,-\,1}$. ${\eta }_{x}$ can be thought of as the probability of error. Thus, we have for these matrices:

1 $$\begin{array}{rcl}R({r}_{j}|{\varepsilon }_{i}) & = & (1-{\eta }_{R}){\delta }_{i,j}+\frac{{\eta }_{R}}{n-1}(1-{\delta }_{i,j}),\\ G({\sigma }_{j}|{r}_{i}) & = & (1-{\eta }_{G}){\delta }_{i,j}+\frac{{\eta }_{G}}{n-1}(1-{\delta }_{i,j}),\\ C({r}_{j}|{\sigma }_{i}) & = & (1-{\eta }_{C}){\delta }_{i,j}+\frac{{\eta }_{C}}{n-1}(1-{\delta }_{i,j}).\end{array}$$

Here *δ*_*i*,*j*_ is a delta function which is one if $i=j$ and zero otherwise.

### Decision making mechanism_

As a result of observation and communication, an individual *i* collects a set of representations ${{\boldsymbol{r}}}_{i}=\{{r}_{i},\{{r}_{ij}\}\}$. Here, *j* includes all the neighbors of *i*, from whom *i* has received a signal, and *r*_*i*_ refers to the observation of the individual *i*, in case it has made an observation. ***r*** can be thought of as an individual’s internal state, which is composed of all the representations an individual has reached, through observation or communication. Individuals need to infer the environmental state based on their internal state. For this purpose they use an inference scheme, or a decision rule. We consider a simple decision rule, a majority rule. In this decision rule, each individual chooses the representation which has happened the highest number of times. This is the belief or inference of the individual about the environmental state.

### Dynamics of the model_

We assume that an environmental state lasts for *T* time steps, such that individuals can make observations and communicate in each time step. At each time step, with probability *h*, each individual makes an observation of the environment (which is in state $\varepsilon$), and reaches representation *r* with probability $R(r|\varepsilon )$. In addition, it possibly receives signals from its neighbors and comprehends the signals using $C(r|\sigma )$ as referring to representation *r*. Reaching internal state ***r***, which is composed of all the representations an individual has collected, the individual forms a belief *b*, given its internal state ***r*** using its decision rule. Finally, it produces a signal *σ* according to $G(\sigma |r=b)$ and transmits it to its neighbors on the communication network. We consider a synchronous update of the network. That is at each time step, all the individuals make an inference based on their internal state and reach a belief *b*, and communicate their beliefs to their neighbors by producing a signal according to $G(\sigma |r=b)$, at the same time. The dynamics repeat in the same way for *T* time steps. Starting from time 1, no individual has a belief until it makes an observation or receives signals.

### Variables of interest and nomenclature_

The collective information acquisition capability of the population can be measured by the fraction of individuals who infer the correct environmental state. We call this the *inference capability*. Another variable of interest to us is the size of the majority group, defined as the largest fraction of population who share the same belief. That is the number of individuals with belief *b*_*m*_, such that for any belief *b*, $N({b}_{m})\ge N(b)$, divided by the population size *N*, $m({\eta }_{R},{\eta }_{G},{\eta }_{C})=\frac{N({b}_{m})}{N}$. Here, *N*(*b*) is the number of individuals with belief *b*.

As explained in the Results section, the model has three phase, informed consensus, uninformed consensus and the disordered phase. As the order parameter of the model we need a variable which distinguishes these three phases. In the following, we will consider two network structures, a first and a second nearest neighbor network both with periodic boundary condition. On a first nearest neighbor network, the size of the majority group takes values close to 1, 0.5, and a small value of the order of $\frac{1}{N}$ in respectively, the informed, uninformed and the disordered phases. Consequently, it serves as the order parameter of the model. The reason why the size of the majority group is close to 0.5 in the uninformed consensus, is that, as a bipartite network is composed of two independent subnetworks, individuals on each subnetwork signal only to those on the other subnetwork. Consequently, the state of each subnetwork is independent of the state of the other subnetwork at the same time and is influenced by the state of the other subnetwork at an earlier time step. This leads to the fact that in the uninformed consensus phase, the two subnetworks form consensus on independent beliefs and the size of the majority group equals the size of the largest subnetwork, which equals 0.5 in our case. [See Supplementary Information, section SI.3. for more details].

However, on a non-bipartite network, such as a second nearest neighbor network, the size of the majority group is a large value close to 1 in both informed and uninformed consensus phases and thus this can not distinguish these two phases. As the order parameter of the model on a general network, we define $\mu ={(-1)}^{(-\nu +1)}m$. Where $\nu$ is equal to one if the majority belief is the same as the environmental state, and zero otherwise. This will allow us to distinguish true from false consensus. For a population which is equipped with a collective sensing system, given by the noise parameters ${\eta }_{R}$, ${\eta }_{G}$, and ${\eta }_{C}$, we denote the inference capability, majority size and the variable *μ*, respectively by ${\rm{\Lambda }}({\eta }_{R},{\eta }_{G},{\eta }_{C})$ and $m({\eta }_{R},{\eta }_{G},{\eta }_{C})$, $\mu ({\eta }_{R},{\eta }_{G},{\eta }_{C})$.

## Results

### Phase digram and the nature of the phase transitions

We run two sets of simulations. We consider a level of noise $\eta$, and in the first simulation, put it in the production matrix, taking the comprehension matrix perfect (noiseless). In the second simulation, we put the noise in the comprehension matrix, making the production perfect. To begin with, we set the representation noise equal to ${\eta }_{R}=0.75$. We consider a population on a 10 × 10 square lattice with first nearest neighbor interaction and periodic boundary conditions. We set the number of environmental states *n* equal to 100. Later, we will show the validity of our results for other parameter values.

**Figure 1:**
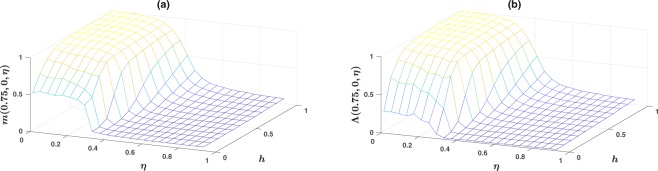
(**a**) The size of majority group $m(0.75,0,\eta )$, and (**b**), the inference capability ${\rm{\Lambda }}(0.75,0,\eta )$, when an amount of noise $\eta$ is present in comprehension, as a function of observation probability *h*, and comprehension noise $\eta$. Here, $N=100$ and the communication network is a first nearest neighbor network.

**Figure 2:**
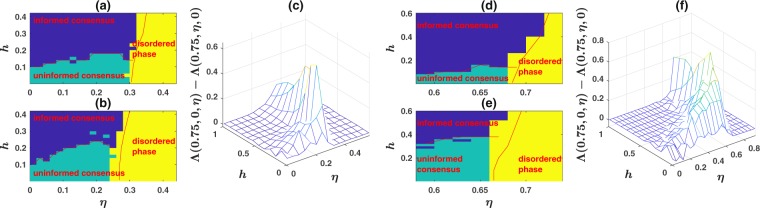
(**a**,**b**) The phase diagram of the model on a first nearest neighbor network, for respectively, the cases when the noise is in comprehension, and when it is in production. The model shows two ordered phases; an informed consensus phase for high observation probability, separated by a first order transition from an uninformed consensus phase. By increasing the noise, the model goes to the disordered phase in which no consensus is formed. Transferring noise from comprehension to production shifts the order-disorder transition line to smaller noise levels and higher observation probabilities *h*. This shows that the production noise is more detrimental for the collective inference of the population. (**c**) The comprehension-production asymmetry, defined as ${\rm{\Lambda }}(0.75,0,\eta )\,-\,{\rm{\Lambda }}(0.75,\eta ,0)$, as a function of *h* and $\eta$, on a 10 × 10 first nearest neighbor network. The shifts in the transition lines lead to the positivity of the asymmetry. (**d**,**e**) The phase diagram of the model on a second nearest neighbor network, for respectively, the cases when the noise is in comprehension, and when it is in production. (**f**) The comprehension-production asymmetry, ${\rm{\Lambda }}(0.75,0,\eta )\,-\,{\rm{\Lambda }}(0.75,\eta ,0)$, as a function of *h* and $\eta$ for a second nearest neighbor network.

We begin by studying the stationary behavior of the system. To do this, we run a simulation for a long enough time *T*, for the system to reach a stationary state and plot the majority size $m(0.75,0,\eta )$, and the inference capability ${\rm{\Lambda }}(0.75,0,\eta )$, as a function of the observation probability *h*, and the noise level $\eta$, respectively in Fig. 1a,b. Here, $T=1500$ and an average over a sample of 200 simulations is taken. For details of simulations see section Methods below. The majority size and the inference capability when the noise is in production is qualitatively similar and not shown. However, as we will discuss below, there is an important quantitative difference in the location of phase transitions. For large noise levels, information transfer is degraded by communication noise and the population fails to reach consensus. By reducing the noise, the system shows a phase transition to an ordered phase in which the population forms consensus on a belief. However, interestingly, the ordered phase itself is composed of two different phases. For high observation probabilities *h*, the information entered to the population through direct observation by individuals is high enough for the population to form a consensus on the correct environmental state. We call this phase *informed consensus*. As the observation probability decreases, the system shows a phase transition to an *uninformed consensus* phase, in which consensus between individuals is formed, however, on a random belief, chosen independently from the environmental state. The phase diagram of the model, for a first and a second nearest neighbor network, when the noise is in comprehension is depicted, respectively in Fig. 2a,d. Here, the two phase transition lines, informed-uninformed, and order-disorder transitions are plotted. As explained below both these transitions are discontinuous. Furthermore, we explain how we determine these transition lines.

**Figure 3:**
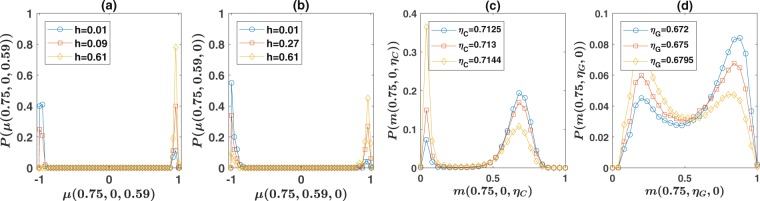
The nature of phase transitions. (**a**,**b**) The distribution of the order parameter *μ* derived from $R=100$ runs. In (**a**) the noise is comprehension noise and in (**b**) it is production noise. In the ordered phase, in each run the system goes to one of the two ordered phases. Consequently, the probability distribution of the order parameter has two peaks each corresponding to uninformed or informed consensus phases. By increasing *h* at fixed $\eta$, the probability of going to the informed consensus increases, while the probability of going to uninformed consensus phase decreases. These show that the informed-uninformed transition is discontinuous. (**c**,**d**) The distribution of the majority size *m* in the order-disorder transition region, derived from a time series of the system, for comprehension noise (**c**), and production noise (**d**). The distribution is bimodal in both cases. By increasing the noise, the peak corresponding to the ordered phase decreases, while that corresponding to the disordered phase increases. This indicates a discontinuous transition.

In the ordered phase, in each simulation the system goes to one of the two ordered phases, that is informed or uninformed consensus. This leads to the fact that the probability distribution of the order parameter *μ* has two peaks, each corresponding to one of the ordered phases. This can be seen in Fig. 3a,b, for respectively the cases that the noise is comprehension noise, and when it is production noise, for the population living on a second nearest neighbor network of size $N=100$, whose phase diagram is presented in Fig. 2d,e. Here, the probability distribution of the order parameter at fixed $\eta =0.59$, which lies in the ordered phase, and different *h*s is plotted. By increasing *h*, the probability that the system goes to the informed consensus increases, while the probability that the system goes to uninformed consensus decreases. This again can be seen in Fig. 3a,b, and is reminiscent of a first order transition^19^. The phase boundary in Fig. 2 is defined as the region in the phase space where the system goes to one of the two phases with a probability close to $\frac{1}{2}$^19^. [See Supplementary Information, section SI.2. for more details].

The nature of the order-disorder transition is investigated in Fig. 3c,d, for respectively, comprehension, and production noise. Here, we consider a population of size $N=100$ on a second nearest neighbor network and take the observation probability equal to $h=0.2$ and derive the distribution of the majority size from a single time series of the system for different values of noise, close to the order-disorder transition. As can be seen, close to the order-disorder transition, the distribution is bimodal. By increasing the noise, the peak corresponding to the ordered phase decreases, while the peak corresponding to the disordered phase increases. This suggests that the order-disorder transition is discontinuous in this case. The order-disorder transition lines in Fig. 2a,b,d,e are determined by locating the noise level where the area below the two peaks approximately equal, for different observation probabilities.

Although both informed-uninformed and the order-disorder transitions are discontinuous, there are differences between the two. Close to the order-disorder transition noise level, the system shows intermittency between the two phases in a single run. [See the Supplementary Information, Fig. SI.2 for an example of the time series of the system]. This is not the case for the informed-uninformed transition, in which in each run the system goes to one of the two ordered phases.

### The comprehension-production asymmetry

The phase diagram of the system when the noise is transferred to production is plotted in Fig. 2b for a first, and in 2e, for a second nearest neighbor network. We see that the phase diagram of the model when noise is in comprehension, and when it is in production are qualitatively similar. However, the order-disorder transition noise level for the same observation probability is shifted to smaller noise levels for production noise. This shows that noise in production degrades the flow of information more severely than noise in comprehension. Consequently, the transition from the consensus phase to the disordered phase occurs at a smaller noise level for production noise. Furthermore, production noise is more detrimental for informed consensus such that the line of first order informed-uninformed transition is shifted to larger values for a noisy production, compared to noisy comprehension. This again shows that production noise is more disruptive for information flow between the individuals and means that, with a noisy production, the population needs a higher net information flow to the population via direct observations to extract environmental state.

To see how this shift affects the inference capability, we define the comprehension-production asymmetry as the difference between the inference capabilities in the case that an amount of noise is in comprehension with that when the same noise is transferred to production, ${\rm{\Lambda }}(0.75,0,\eta )\,-\,{\rm{\Lambda }}(0.75,\eta ,0)$ (in the following we call this quantity asymmetry for short). We plot this in Fig. 2c for the first, and in Fig. 2f, for the second nearest neighbor networks. We see that the shift in the transitions leads to the positivity of this quantity. Each positive region in Fig. 1c,f has a different root. The positive region at, and to the right of the order-disorder transition line results from the fact that the order-disorder transition noise level shifts to smaller values for production noise, while the positive region to the left of the order-disorder transition lines in low observation probabilities results from the fact that production noise shifts the line informed-uninformed transition to larger observation probabilities. This shape is characteristic of the asymmetry and is preserved for different parameter values. As we see, the asymmetry captures the differences in phase diagrams between production and comprehension noise, consequently we will focus on it to study the comprehension-production asymmetry for different parameter values.

### Dependence on the parameters of the model

**Figure 4:**
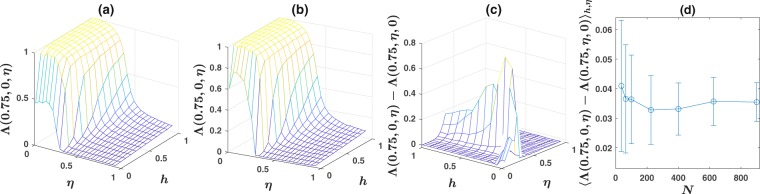
(**a**,**b**) The inference capability ${\rm{\Lambda }}(0.75,0,\eta )$, for a population of size, respectively, $N=400$ and $N=900$, on a first nearest neighbor network. (**c**) The comprehension production asymmetry ${\rm{\Lambda }}(0.75,0,\eta )-{\rm{\Lambda }}(0.75,\eta ,0)$ for a population of size $N=900$, on a first nearest neighbor network. (**d**) The mean asymmetry over *h* and $\eta$, ${\langle {\rm{\Lambda }}(0.75,0,\eta )-{\rm{\Lambda }}(0.75,\eta ,0)\rangle }_{h,\eta }$, as a function of population size *N*. The mean asymmetry slightly decreases with *N* for small population sizes up to $N=200$, and shows small variation with *N* for larger *N*.

We begin our analysis regarding the robustness of our findings with respect to the model parameters, by studying the dependence of the results on the population size. The inference capability for the comprehension noise ${\rm{\Lambda }}(0.75,0,\eta )$, as a function of *h* and $\eta$ for a population of size $N=400$ and $N=900$ on a first nearest neighbor network, is plotted respectively, in Fig. 4a,b. See section Methods for details of simulation in this section. The case of production noise, leads to a similar picture. As can be seen by comparing with Fig. 1b for $N=100$, while the order-disorder transition line shows very small sensitivity with respect to the population size, the informed-uninformed transition line, shows stronger sensitivity to the population size and shifts to smaller *h* values by increasing the population size. This phenomena has an interesting interpretation and means that, for a larger population, a smaller fraction of observant individuals is needed for the group to collectively infer the correct environmental state. A similar result to this finding had been noticed before, and can be argued to be resulted from the wisdom of crowd effect^5^. However, our result gives an interesting aspect to this finding, by reinterpreting it as resulting from a shift in a discontinuous phase transition, due to finite size effects.

The comprehension-production asymmetry ${\rm{\Lambda }}(0.75,0,\eta )\,-\,{\rm{\Lambda }}(0.75,\eta ,0)$ as a function of *h* and $\eta$, for a population of size $N=900$ on a first nearest neighbor network is plotted in Fig. 4c. By comparing with Fig. 2c, it can be seen that, while the asymmetry arising from the shift in the order-disorder transition shows little sensitivity to the population size, That part of asymmetry resulting from the shift in the uninformed-informed transition, shows stronger sensitivity, and decreases by increasing population size and seems to tend to a stationary value for large population sizes.

To see the overall behavior of the asymmetry with respect to the population size, the average of the asymmetry over *h* and $\eta$, ${\langle {\rm{\Lambda }}(0.75,0,\eta )-{\rm{\Lambda }}(0.75,\eta ,0)\rangle }_{h,\eta }$ as a function of *N* is plotted in Fig. 4d. We see that it slightly decreases with population size, for population of size up to $N=200$, and shows small sensitivity to population size for larger populations.

**Figure 5:**
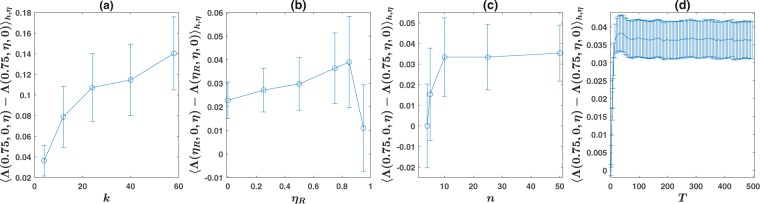
(**a**) The mean asymmetry over *h* and $\eta$, ${\langle {\rm{\Lambda }}({\eta }_{R},0,\eta )-{\rm{\Lambda }}({\eta }_{R},\eta ,0)\rangle }_{h,\eta }$, as a function of network connectivity *k*, defined as the number of neighbors. Here a population of size $N=100$ is set on a lattice with periodic boundary condition and 1st up to 5th nearest neighbor interactions. (**b**) The mean asymmetry as a function of representation noise ${\eta }_{R}$. The mean asymmetry increases with ${\eta }_{R}$ up to high noise levels and drops for very large representation noises. (**c**) The mean asymmetry as a function of the number of states *n*. The mean asymmetry increases with *n* until it saturates to a constant value for large *n*. (**d**) Non-stationary behavior. The mean asymmetry as a function of time *T*. Asymmetry is zero for $T=1$, and rapidly increases and tends to a stationary limit for large times.

We note that the asymmetry increases in situations where communication has stronger effect on the inference by the population. One circumstance where the effect of communication is increased is by increasing network connectivity. In Fig. 5a we plot the average of the asymmetry over *h* and $\eta$, ${\langle {\rm{\Lambda }}(0.75,0,\eta )-{\rm{\Lambda }}(0.75,\eta ,0)\rangle }_{h,\eta }$ as a function of network connectivity *k*, defined as the number of neighbors. In this simulation, we have considered a population of $N=100$ individuals on a square lattice with 1st up to 5th nearest neighbor interactions, and periodic boundary conditions. We have set $n=100$ and $T=500$. We see that the mean asymmetry increases with network connectivity. How this increase comes about can be seen by comparing Fig. 2a,b for a first, with Fig. 2d,e for a second nearest neighbor network. As seen, the shift in first order transition line is much higher for a second nearest neighbor network.

Another circumstance where communication has stronger effect on the inference of the population, is when noise in representation increases. In such cases, communication is more indispensable in providing crucial information for the individuals to remedy their highly noisy observations. By plotting ${\langle {\rm{\Lambda }}({\eta }_{R},0,\eta )-{\rm{\Lambda }}({\eta }_{R},\eta ,0)\rangle }_{h,\eta }$ as a function of ${\eta }_{R}$ in Fig. 5b, we see that the asymmetry increases with representation noise for up to high values of ${\eta }_{R}$. It gradually tends to zero as representation formation tends to become random, as in this case there is no information entered to the population via observation to be amplified by communication.

The behavior of the asymmetry with respect to the number of environmental states *n*, is investigated in Fig. 5c. Here, we note that as ${\eta }_{R}=0.75$, with $n=4$ state, the representation formation is uniformly random. Thus, we expect no asymmetry in this case as there is no information entered to the population via direct observation. By increasing *n*, the asymmetry shows up and increases, until it approaches a stationary value for large *n*.

So far, we have considered the stationary behavior of the system. A curious question is how the comprehension-production asymmetry behaves in the non-stationary dynamics. To see this, we plot ${\langle {\rm{\Lambda }}(0.75,0,\eta )-{\rm{\Lambda }}(0.75,\eta ,0)\rangle }_{h,\eta }$ as a function of environmental duration time scale *T*, in Fig. 5d. As can be seen, the asymmetry increases with *T* until it saturates to a stationary limit. This implies that in a situation where signal transmission time scale is of the same order of environmental duration time scale, $T=1$, there is no or little asymmetry in signal comprehension and production. While in biological communication systems, that operate in a situation where environmental time scale is longer than signal exchange time scale, an asymmetry between signal comprehension and production is observable.

In the Supplementary Information [see Supplementary Information], we consider modifications of the decision rule in which individuals exploit the distinction between representations provided by direct observation, and those provided by communication, by weighting their direct observation with a factor $\omega  > 1$, when making an inference. Such a more self-confident decision rule can be useful in high communication noise regimes. We show that the asymmetry also holds under such modifications of the decision rule (Fig. SI.3). Furthermore, we show the validity of our results for different network structures (Fig. SI.3). In addition, we consider the effect of a background noise added to both comprehension and production faculties, and show that the asymmetry endures in the presence of such background noise as well (Fig. SI.2).

## Discussion

In this paper we analyzed a simple model of communication between agents trying to collectively infer an environmental state. We described the phase diagram of this model and showed that noise in production of signals is more detrimental for the information acquisition capability of a communicating population, compared to noise in comprehension. This means that, assuming biological organisms have limited resources to devote to noise reduction in the communication system, they do better if they devote the resources to noise reduction in production more than in comprehension. Consequently, we conjecture that biological communication systems should have higher levels of regulation on signal production to reduce errors.

In addition, the analysis of our model reveals the conditions over which the comprehension-production asymmetry is stronger, and thus provides hints at where it is more likely for such an asymmetry to be more readily observable in nature. Specifically, our analysis shows that the asymmetry is strongest in populations living in smaller groups, groups with high connectivity, and groups composed of individuals with noisier senors. An example where the first, and probably also the second condition holds, seems to be primate and early human societies^20^. As early humans used to live in small populations of size approximately 100 or less^20^, we conjecture that this asymmetry should be observable in the case of human language. The fact that such an asymmetry in language learning exists has been noticed, for example in the case of comprehension and production of sentence order^21^, and pronouns and reflexives agreement^22^. According to this phenomena, correct production of sentences requiring certain grammatical competence, occurs with higher probability compared to correct comprehension, during language learning in certain ages. Understanding the mechanism behind this asymmetry in language learning is a very interesting problem, in particular given that some theoretical work on the subject suggest that mastery of language comprehension precedes language production^17,23^. Our model provides an evolutionary explanation for this phenomenon: as evolutionarily it is more advantageous to make oneself understandable than to understand, such an asymmetry can be the vestige of an evolutionary advantageous architecture of language. More accurate empirical tests, which are devised to specifically examine comprehension-production asymmetry, for example in the form of language games, can shed more light on the question whether such an asymmetry indeed exists in humans.

Among other examples of collective information acquisition systems where the existence of a comprehension-production asymmetry can be tested, here we discuss honey bee and ant societies. In ant societies, a successful forager by laying a pheromone trail recruits other workers^12^. As a laid trail is accessible to many ants (potentially the whole colony), it can be argued that this communication system has a very high network connectivity. On the other hand, poor performance of individual ants in finding resources seems to suggests ants posses a noisy sensor^24^. Our theory predicts a strong comprehension-production asymmetry in this case. Although it is argued that this communication system is highly noisy^24,25^, how the noise in comprehension compares to noise in production needs to be studied. Another example where comprehension-production asymmetry can be explored is the case of honey bees. Communication between honey bees is performed by waggle dance which conveys information about the direction and distance of a desired source from the hive^12,13,26,27^. As opposed to the case of ants, where the trail can last for hours to be felt by other individuals, the signal in bees is mainly visual and lasts for seconds^26^. It thus can be argued that the connectivity of the communication network in this case, is much smaller than that of ants. Our theory thus predicts a smaller asymmetry in this case.

Here, we suggest an experimental set up to explore whether a comprehension-production asymmetry exists in animal communities. We state the argument having bees in mind as an example, however, a similar experiment can be performed with other species. Assume we record the roots taken by a number, say *n*_1_ bees all of whom has observed a single waggle dance. Denote the path (or the angle of deviation at a distance *x* from the hive, measured with respect to the line joining the hive to the goal in a two dimensional plane) of the *i*th bee by *d*_*i*_. As experiments of this kind has shown, the path taken by the bees is subject to fluctuations^27^. Since all the *n*_1_ bees has observed the same dance, any variation in their paths has to be attributed to noise in each bee’s comprehension of the signal. A measure of the dispersal of the individual paths around the mean path (e.g. standard deviation) of the *n*_1_ bees, could thus be argued to be a measure of comprehension noise (plus possibly noise in decision making). For the case of the standard deviation this can be written as ${\eta }_{C}={\langle {({d}_{i}-{\langle {d}_{i}\rangle }_{i})}^{2}\rangle }_{i}$. Where the subindex *i* indicates an average over the *n*_1_ bees. On the other hand, assuming errors in bees’ comprehension is distributed symmetrically around zero, and *n*_1_ is large, the mean of the path over all the *n*_1_ bees who has observed the same signal, gives the signaled direction. Now assume we perform such an experiment for *n*_2_ times. Denote the mean path of the bees in the experiment *α* by *d*_*α*_, that is ${d}_{\alpha }={\langle {d}_{i}\rangle }_{i}$ in experiment *α*. Now consider a measure of the dispersal, say standard deviation, of *d*_*α*_ around its mean over *n*_2_ experiments, ${\eta }_{G}={\langle {({d}_{\alpha }-{\langle {d}_{\alpha }\rangle }_{\alpha })}^{2}\rangle }_{\alpha }$. Assuming the cost associated with noise reduction in comprehension and production are more or less the same, our theory predicts that ${\eta }_{G}\le {\eta }_{C}$. In principle, similar experiments can be performed with other species to test the comprehension production asymmetry.

Another interesting prediction of our model is that biological communication systems, in the presence of some level of noise in communication, up to a threshold ${\eta }^{\ast }$, perform almost as well as a noiseless communication system. This results from the fact that the transition to the ordered/high inference capability phase happens at a non-zero noise level. This means that, since a marginally noisy communication system performs almost as well as a noiseless communication system, there is little selection to reduce noise, once in the small noise region.

Although it is argued that information sharing increases accuracy of decision making^28–30^, it has been noticed that the same phenomena can lead to pathological effects such as herding on an inferior decision or formation of a wrong belief^31^. This can be the case, in situations where individuals have no way of judging the quality of the information provided by their group mates. As experimental work has demonstrated, in such cases, relying on social information can lead to false consensus^32^. It is argued, for example, that financial crashes are results of such collective decision making failure by herding on an inferior choice^33^. Our model provides a theoretical framework to explain and interpret this phenomena in terms of an uninformed-informed phase transition, and shows that for collective decision making to be successful, there has to be enough information flow to the population through direct observations by individuals, otherwise the system will be stuck in an uninformed consensus phase.

In addition, the finite size analysis of our model reveals that the fraction of observant individuals needed for the group to correctly infer the environmental state decreases with the population size^5^, due to a shift in the uninformed-informed transition to smaller observation probabilities by increasing the population size.

The model introduced here has profound similarities with ferromagnetic systems in statistical physics^34^. In both, the signaling system introduced here, and magnetic systems such as Ising/Potts models, a set of agents, who can take one out of *n* possible states, are coupled with one of the states with an external field, and try to align themselves through an interaction term, in a noisy background. The equivalent of the external field in our model is the probability of faithful observation of the environment. The interaction in our model is provided with signaling between agents and their efforts to align their beliefs with each other according to the signals they receive. In fact, the two ordered phases, uninformed and informed consensus phases, can be thought of as analogs of spin up and spin down ordered phases in ferromagnetic systems, which are separated by a first order transition in both cases. The order-disorder transition however, as shown, has a different phenomenology in our model of collective information acquisition, compared to the Ising model: while in the Ising model the order-disorder transition happens at a single critical point, our model posses a line of discontinuous order-disorder transition. Another important fundamental difference is that, while in ferromagnetic systems there is only one type of noise, the thermal noise, in this model of communication, we have two different types of noise which operate differently. Comprehension noise acts independently in each signal exchange event. Production noise however, by affecting a signal from the source, effectively acts in a more correlated fashion.

Regarding the similarity of our model with Ising and Potts models, a future direction of research can be to investigate this similarity in more depth. In fact, it is possible to formulate a similar model in terms of Potts variables by assigning to each agent, three Potts variables for signals, representation and belief, which are represented as binary vectors of length *n*, where *n* is the number of possible states. For example, we can represent agent i’s belief by ${\overrightarrow{b}}_{i}=(0,\mathrm{..},0,1,0,\mathrm{..0})$, where a 1 in *a*th entry means agent *i* believes in state *a*. Assuming these variables obey Boltzmann statistics, it is possible to write expressions for their means. Imposing a self consistency requirement, which equals the variables with their means, leads to mean field equations for equilibrium distribution of the variables [Hertz, J. Salahshour, M. and Roudi, Y. under preparation].

The effect of network structure on the inference of the population can be another avenue for future research. Although, the effect of network structure on consensus formation and spread of opinions has attracted much attention^35–38^, but less attention has been payed to the effect of network structure on the inference capability of the population. Our model, by distinguishing the two informed and uninformed consensus phases, provides an interesting mathematical framework for the study of the effect of network structure on true consensus formation and the inference capability of the population. For example, applying the dynamics on real world sensory networks^38,39^ seems to provide valuable insights into the beneficial aspects of real world sensory networks for collective inference of the population. For instance, as already can be seen here, higher network connectivity has a double sided effect. On the one hand it facilitates the ordering process and shifts the order-disorder transition to larger noise levels, and on the other hand it can promote uninformed consensus by shifting the informed-uninformed transition line to larger noise levels. How communicating populations manage to benefit from higher network connectivity and at the same time avoid its detrimental effects can be an interesting question to investigate both theoretically and empirically.

## Methods

### Dynamics of the model

As explained in the section The Model, the dynamics of the model is as follows. The population is set on a communication network, such that each individual communicates with its neighbors on the network. We consider a synchronous update of the network. That is at each time step, each individual makes an observation with probability *h*, and reaches representation *r* according to $R(r|\varepsilon )$. Besides, the individual possibly receives a set of signals from its neighbors and transforms each signal to a representation, according to $C(r|\sigma )$. Reaching internal state ***r***, which is the set of all the representations an individual has received, it forms a belief *b* using its decision rule, given its internal state ***r***, and communicates its belief to its neighbors by producing a signal according to $G(\sigma |r=b)$. Starting from time 1, no individual has a belief to communicate, until it makes an observation or receives a signal.

### Simulations

The basic parameter values used in the simulations are presented in Table 1. In each simulation some parameters are changed as explained in the figures. An average over *R* runs is taken to calculate the variables of interest. In Figs 1a,b and 2c,f, *R* is set equal to 200. For the simulations used to calculate phase diagrams in Fig. 2a,b,d,e, which is the same simulation used in Fig. 3a,b, $R=100$. The distributions of the majority size presented in Fig. 3c,d is calculated based on a single time series of length $T={10}^{5}$, after discarding the first 10^4^ steps, for a population of size $N=100$ and on a second nearest neighbor network. To calculate the graphs in Figs 4 and 5, the same parameter values as in Table 1 are used. However, in Figs 4 and 5a–c, $R=24$, and in Fig. 5d, $R=200$. In Figs 1, 4 and 5, $T=500$.

**Table 1:** The basic parameter values used in the simulations.

Parameter	Value
*L*	10
*N*	100
*n*	100
*T*	1500

The population resides on a *L* × *L* lattice. *N* is the population size, *n* is the number of states, *T* is time.

## Supplementary information

LaTeX Supplementary File

Supplimentary Information pdf file
