# Supplementary material for: Phase transitions and asymmetry between signal comprehension and production in biological communication
Source: Sci Rep. 2019 Mar 5;9:3428. doi: 10.1038/s41598-019-40141-4 (PMC6401316; doi:10.1038/s41598-019-40141-4)
Supplement: Supplementary file 2 — Supplimentary Information pdf file [file 41598_2019_40141_MOESM2_ESM.pdf]

Supplementary Information for phase transitions and asymmetry between signal comprehension and production in biological communication.

M. Salahshour, S. Rouhani,
Department of Physics, Sharif University of Technology, P.O.Box 11155-9161, Tehran, Iran
Y. Roudi
Kavli Institute for Systems Neuroscience and Centre for Neural Computation, Norwegian University of Science and Technology (NTNU), Olav Kyrres gate 9, 7030 Trondheim, Norway

SI. 1. SIMULATIONS

The basic parameter values used in the simulations presented in the Supplementary Information in the following are the same as those in the main text and are reported in Table. (I). In each simulation some parameters are changed as explained in the figures. An average over R runs is taken. In Fig. (SI.2.b), Fig (SI.3.c), Fig (SI.3.d), $R = 200$, and for the simulations used to derive the graph in Fig. (SI.3.a), and the one in Fig. (SI.3.b), $R = 24$. Finally, the simulations used in Fig. (SI.1), are the same set used to derive the phase diagram in Fig. (2) in the main text, for which $R = 100$.

parameter	value
L	10
N	100
n	100
T	1500

TABLE I. The basic parameter values used in the simulations. The population resides on a $L \times L$ lattice. N is the population size, n is the number of states, T is time.

SI. 2. UNINFORMED-INFORMED TRANSITION AND THE PHASE DIAGRAMS

As expected, near the line of first order transition, in each simulation, the system goes to one of the two, informed or uninformed consensus phases. The probability that the system goes to each one of these phases are plotted in Fig. (SI.1) as a function of h and η. These are calculated for a population of size $N = 100$ living on a first nearest neighbor square lattice with periodic boundary conditions. As we see in Fig. (SI.1.a) for the case when the noise is in comprehension, and Fig. (SI.1.c) for the case when the noise is in production, by increasing the observation probability h, the probability that the order parameter takes the value $m = \frac{1}{2}$, which corresponds to the uninformed consensus phase, decreases. On the other hand, as can be seen in Fig. (SI.1.b) and Fig. (SI.1.d), for respectively the cases when the noise is in comprehension and in production, the probability that the order parameter takes the value $m = 1$, which corresponds to the informed consensus phase, increases by increasing h. This phenomenology is characteristic of a first order transition [20]. The line of first order transition in Fig. (2.a) and Fig. (2.b) in the main text, is defined as the region where these probabilities equal $\frac{1}{2}$. In practice, near the order-disorder transition, the order parameter does not take the exact value expected for the corresponding phase (1 for informed, and $\frac{1}{2}$, for uninformed consensus phase) due to statistical fluctuations. Consequently, we considered a region to belong to the informed consensus (dark blue) if $P(0.7 < m) > 0.5$ and $P(m > 0.7) + P(0.4 < m < 0.6) > 0.9$. Equivalently, a region is considered to belong to uninformed consensus (green) if $P(0.4 < m < 0.6) > 0.5$ and $P(m > 0.7) + P(0.4 < m < 0.6) > 0.9$. Otherwise it is indicated to belong to the disordered phase (yellow). Changing these values does not change the phase diagrams significantly. In Fig. (2.d) and Fig. (2.e) the same procedure is used. Here, a region is indicated to belong to the informed consensus (dark blue) if $P(0.6 < m) > 0.5$ and $P(m > 0.6) + P(m < -0.6) > 0.8$. Equivalently, a region is considered to belong to uninformed consensus (green) if $P(m < -0.6) > 0.5$ and $P(m > 0.6) + P(m < -0.6) > 0.8$. Otherwise it is indicated to belong to the disordered phase (yellow). Changing these values does not change the phase diagrams significantly.

The line of first order order-disorder transitions in Fig. (3.a) and Fig. (3.b), for a first, and Fig. (3.d) and Fig. (3.e) for a second nearest neighbor network, are determined in this way. For each observation probability h, we derive the

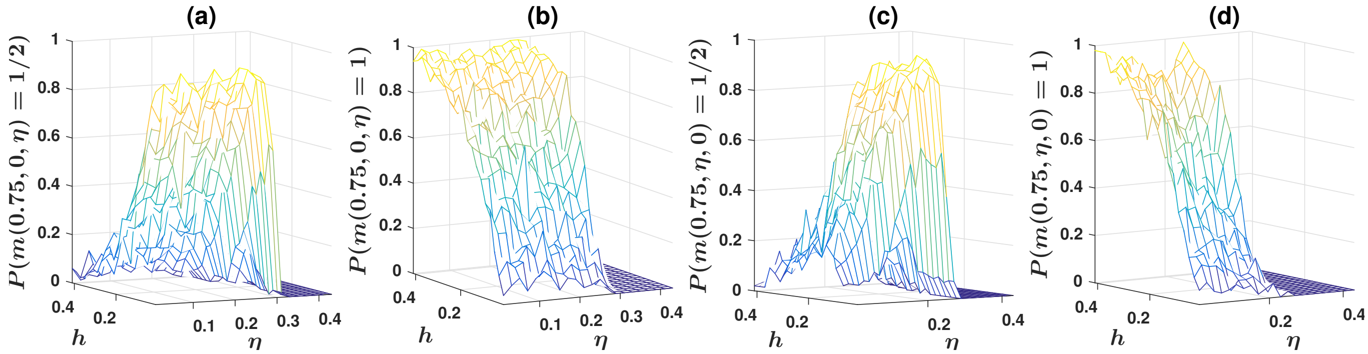

FIG. SI.1. (a) and (b), the probabilities that the order parameter $m(0.75, 0, \eta)$, takes the values, respectively, $m = \frac{1}{2}$, and $m = 1$. (c) and (d), the probabilities that the order parameter $m(0.75, \eta, 0)$, takes the values, respectively, $m = \frac{1}{2}$, and $m = 1$. We see that for η less than an order-disorder transition level, for low observation probabilities the system goes to the uniformed consensus phase with high probability, and for high observation probability, the system goes to the informed consensus phase with high probability. The line of first order transition is defined as the line where these probabilities equal $\frac{1}{2}$.

distribution of the majority size, from a single time series for different ηs in the order-disorder transition region. As can be seen in Fig. (SI.2.a), such time series show intermittency between the two phases, and thus their distribution has two peaks, each corresponding to one of the phases. The transition noise level for each h, $\eta(h)$, is determined as the noise level where the area below the peaks corresponding to different phases approximately equal. Due to the fact that this transition is very sharp, we are able to determine the transition noise level with high accuracy in this way.

SI. 3. THE SPATIO-TEMPORALLY BLINKING PHASE ON A BI-PARTITE NETWORK, AND THE CHOICE OF THE ORDER PARAMETER

In this section, we intend to explain the choice of the order parameter. The order parameter needs to distinguish the three, uninformed, informed and disordered phases. For a $L \times L$, nearest neighbor network with periodic boundary condition with L even, the size of the majority group normalized by the population size, defined as $m = \frac{N(b)}{N}$, takes the values $\frac{1}{2}$, 1, and a small value of the order of $\frac{1}{N}$, in respectively, the uninformed, the informed, and the disordered phases. Consequently, it distinguishes the three phases and can serve as the order parameter of the model. The reason why the size of the majority group is $\frac{1}{2}$ in the uninformed consensus phase on such a network is explained below.

On a bipartite network (i.e. a network which can be divided to two independent sub-networks, such that the vertices of each sub-network are disconnected and only connected to the vertices from the other sub-network), such as an $L \times L$ first nearest neighbor lattice with periodic boundary condition, with L even, in the uninformed consensus phase, the individuals on each independent sub-network form the same belief, which is independent of the belief in the other sub-network. As the two sub-networks are related by signaling, the state of the two networks is interchanged in each time step. Consequently, this phase can be thought of as a spatio-temporally blinking phase (In general we expect a similar phenomena happens on an n-partite network). The reason is that the belief of individuals at time t, is determined by the belief of the individuals on the other sub-network at time $t-1$. Consequently, individuals on each sub-network form the same belief which is the same as the belief of the individuals on the other sub-network at one earlier time step. Therefore, in the uninformed consensus phase the individuals form two groups each of size $\frac{1}{2}$, and the size of the majority group, m, takes the value $\frac{1}{2}$. While in the informed consensus phase, all the individuals form the same belief, which is the same as the environmental state, and thus $m = 1$.

However, this is the case only for a square $L \times L$ first nearest neighbor lattice with periodic boundary condition, only if L is even. For an $L \times L$ first nearest neighbor lattice with periodic boundary condition and L odd, or in the case of a network with up to kth nearest neighbor interaction, with $k > 1$, the network can not be divided into two separate sub-networks and consequently in the uninformed consensus phase, all the individuals assume the same belief which does not coincides with the environmental state. Consequently, the size of the majority group would become 1 in both phases and this can not serve as a proper order parameter on such networks. To attain a proper order parameter which distinguishes the two phases, we define the order parameter for a second nearest neighbor network as $\mu = (-1)^{(-\nu+1)} \frac{N(b)}{N}$. Where, $\nu = 1$ if the belief of the majority group is the same as the environmental state, and zero otherwise. $N(b)$ is the number of individuals in the majority group and N is the population size. This variable takes the values -1, 1 and a small value of order $\frac{1}{N}$, in respectively the uninformed, informed and disordered phases,

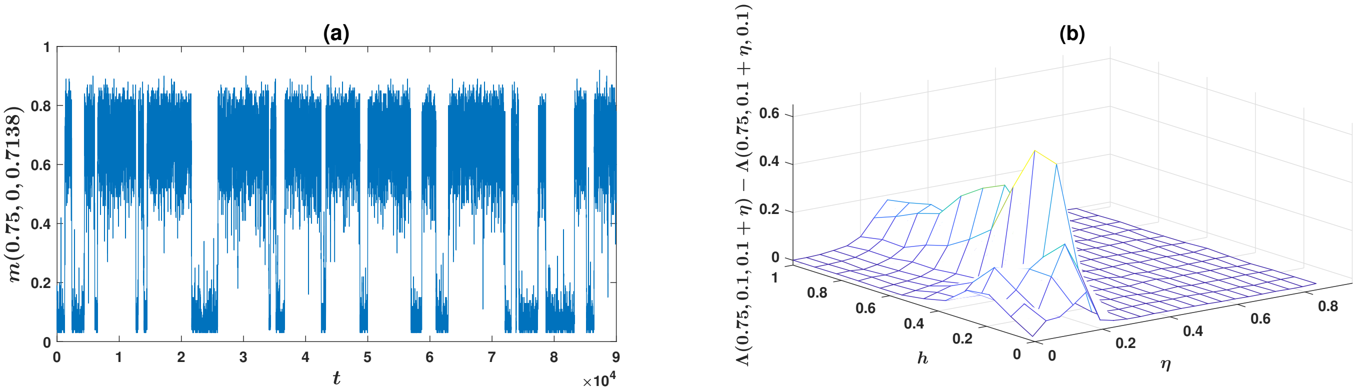

FIG. SI.2. (a) An example of the time series of the system close to the order-disorder transition. Here, $h = 0.2$ and the communication network is a 10×10 second nearest neighbor lattice. (b) The asymmetry $\Lambda(0.75, \eta_0, \eta_0 + \eta) - \Lambda(0.75, \eta_0 + \eta, \eta_0)$ in the presence of a background noise $\eta_0 = 0.1$. The asymmetry shows the same behavior as in the absence of a background noise; It remains positive. This results from shift in the transition points by transferring noise from comprehension to production.

and thus, can distinguish the three phases and serves as a proper order parameter.

SI. 4. THE EFFECT OF BACKGROUND NOISE

Here, we check that if the asymmetry between comprehension and production noise discussed in the main text endures in the presence of a background noise. We add a background noise η_0 to both comprehension and production channels, and compare the effect of addition of an extra amount of noise η to one of them. We plot $\Lambda(\eta_R, \eta_0 + \eta, \eta_0) - \Lambda(\eta_R, \eta_0, \eta_0 + \eta)$ setting $\eta_0 = 0.1$ and $\eta_r = 0.75$, in Fig. (SI.2.b). Here, we see the presence of a background noise does not alter the behavior of the asymmetry. It remains positive, due to the shifts in the phase transitions.

SI. 5. THE EFFECT OF DIFFERENT CONFIDENCE LEVEL ON DIRECT OBSERVATIONS AND COMMUNICATION

In general, in such communicating populations that we consider, there is a distinction between representations reached through direct observation and through communication. This triggers the question whether it is wiser for an agent to rely on its direct observation more, or on the information reached through communication? For example for high communication noise and low representation errors, a good decision making strategy is the one which weighs the representations reached through direct observation more. On the opposite regime, high representation noise and low communication noise, such that the population is in the informed consensus phase and communication conveys information, a good strategy puts lower weight on direct observation. We can take this distinction between representations reached through observation and communication into account, by considering modifications of the majority decision rule with different weights for observational representations and communicational representations. That is, when counting the representations in its internal state, an agent uses a weight factor ω for its observational representations. A simple majority rule is the one with $\omega = 1$. As the population is residing on a square lattice with periodic boundary conditions and first nearest neighbour interactions, each individual has 4 neighbors and receives at most 4 signals. Consequently, $\omega = 4 + \delta$, where δ is a positive number, means that individuals rely on communication, only in the case they do not make an observation. This is the extreme case of highly self-confident agents. $\omega = 1$ reflects the other extreme, where agents treat observational and communicational representations on equal footings. To investigate the effect of different self-confidence levels, we run simulations with different self-confidence levels, $\omega = 1$ to $\omega = 5$. The inference capability, $\Lambda(0.75, 0, \eta)$, for $\omega = 2$ is plotted in Fig. (SI.3.b). From Fig. (1.b) in the main text we see that for $\omega = 1$, in the high communication noise regime, the inference capability drops to a low value, even for high observation probabilities. This shows that communication is detrimental in this high noise regime and the individuals are better off not taking the communication into account. This can be seen by comparing to the case with $\omega = 2$ in Fig. (SI.3.b), where by putting more weight on direct observation if made any, for high communication noise regime, the population remains better off. However, for low communication noise levels higher self-confidence

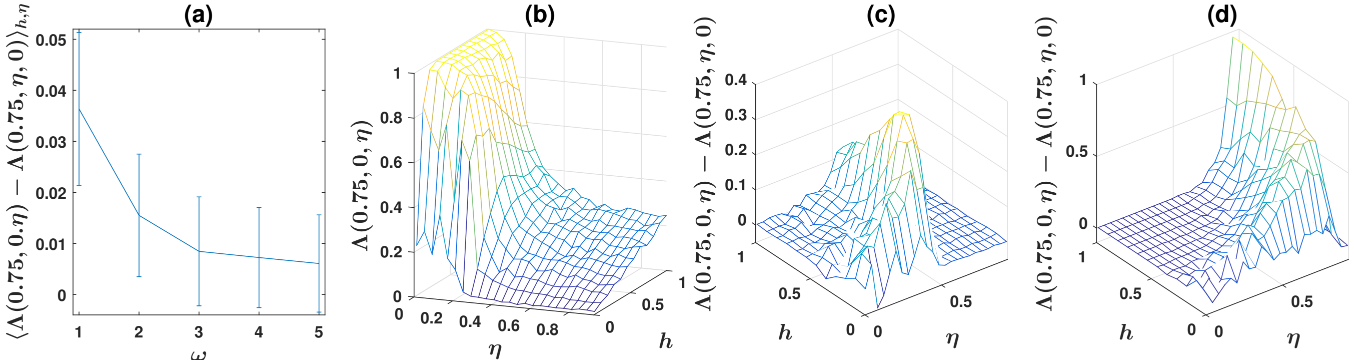

FIG. SI.3. The effect of self confidence. Agents use a modified majority rule as their decision rule, in which direct observation is multiplied by a factor of ω when counting the representations. (a) The average asymmetry over h and η as a function of ω. A more self-confident decision rule (larger ω), by being less reliant on communication, decreases the asymmetry. (b) The inference capability $\Lambda(0.75, 0, \eta)$, when the noise is in comprehension, and with a decision rule with $\omega = 2$. We see that the effect of a higher confidence on direct observation is a shrinking of the informed consensus phase (to be compared with Fig. (1.b) in the main text for $\omega = 1$). However, the advantage of a higher confidence on direct observation is apparent in the high communication noise regime where it increases the inference capability proportionally with observation probability. The reason is that in the disordered phase, communication is information-less and ignoring the communicational signals increases the inference capability. (c) and (d), The effect of network structure. The asymmetry $\Lambda(0.75, 0, \eta) - \Lambda(0.75, \eta, 0)$, as a function of η and h, for a population of size $N = 100$, on a Barbasi-Albert network with mean degree 4 (c), and on a fully connected network (d). We see that the asymmetry is positive on both networks. This shows the same shifts in transition points by transferring noise from comprehension to production.

can be harmful. As it can be seen it results in a shrinking of the informed consensus phase (to be compared with Fig. (1.b) in the main text for $\omega = 1$). This is because, In the ordered phase, communication, by amplifying information entered through observations made by different individuals, increases the accuracy. Consequently, collective belief is a more accurate estimate of the environmental state compared to direct observations.

In Fig. (SI.3.a) we return to the asymmetry by plotting $\langle\Lambda(0.75, 0, \eta) - \Lambda(0.75, \eta, 0)\rangle_{h,\eta}$ as a function of ω. We see that the asymmetry decreases with increasing ω. This should be obvious, as a more self-confident decision rule makes the individuals less reliant on communication. Consequently, the asymmetry, being a phenomenon rooted in communication, becomes less evident, although still existent, for larger ωs.

SI. 6. THE EFFECT OF NETWORK STRUCTURE

In the studies presented so far, we have fixed the network structure to a square lattice. Here, we investigate the effect of network structure and check that our results are valid on different network structures. We consider two different communication network structures. The first is a scale free Barbasi-Albert network of size $N = 100$ and mean degree equal to 4. The second network we consider is a fully connected network of size $N = 100$, in which the whole population communicate with each other. We set a population of size $N = 100$ on each of these networks and calculate the comprehension-production asymmetry. The parameters of the simulations are presented in Table. (I). An average over $R = 200$ simulations is taken. In Fig. (SI.3.c) and Fig. (SI.3.d), we plot the comprehension-production asymmetry for respectively, the Barbasi-Albert network and fully connected network. We see that the asymmetry is positive for both networks. More specifically its shape resemble the shape of the asymmetry on a regular lattice; the positive region in low observation probabilities, results from the shift in the first order transition line between informed and uninformed consensus phases, and the positive region to the right of the diagrams results from the shift in the order-disorder transition point.
